# Connectivity and Topology Invariance in Self-Assembled and Halogen-Bonded Anionic (6,3)-Networks

**DOI:** 10.3390/molecules22122060

**Published:** 2017-11-24

**Authors:** Franck Meyer, Tullio Pilati, Konstantis F. Konidaris, Pierangelo Metrangolo, Giuseppe Resnati

**Affiliations:** Laboratory of Nanostructured Fluorinated Materials (NFMLab), Department of Chemistry, Materials, and Chemical Engineering “Giulio Natta”, Politecnico di Milano, Via L. Mancinelli 7, 20131 Milano, Italy; franck.meyer@ulb.ac.be (F.M.); tullio.pilati@gmail.com (T.P.); konstantis.konidaris@polimi.it (K.F.K.); Pierangelo.metrangolo@polimi.it (P.M.)

**Keywords:** halogen bonding, supramolecular chemistry, crystal engineering, anion coordination

## Abstract

We report here that the halogen bond driven self-assembly of 1,3,5-trifluorotriiodobenzene with tetraethylammonium and -phosphonium bromides affords 1:1 co-crystals, wherein the mutual induced fit of the triiodobenzene derivative and the bromide anions (halogen bond donor and acceptors, respectively) elicits the potential of these two tectons to function as tritopic modules (6,3). Supramolecular anionic networks are present in the two co-crystals wherein the donor and the acceptor alternate at the vertexes of the hexagonal frames and cations are accommodated in the potential empty space encircled by the frames. The change of one component in a self-assembled multi-component co-crystal often results in a change in its supramolecular connectivity and topology. Our systems have the same supramolecular features of corresponding iodide analogues as the metric aspects seem to prevail over other aspects in controlling the self-assembly process.

## 1. Introduction

Non-covalent intermolecular interactions hold a major role in the design of supramolecular assemblies and hence constitute a critical tool for crystal engineering [1]. Among the various intermolecular interactions, halogen bonding (XB) has attracted considerable interest in recent years [2], and systems self-assembled under XB control have found applications in several areas, spanning biopharmacology [3,4,5], catalysis [6,7], and materials science [8,9,10]. XBs display similar characteristics with hydrogen bonds (HBs) in terms of their strength and high directionality [2]. 

XB directionality plays a particularly critical role in the design and synthesis of supramolecular systems as it translates tectons geometry into self-assembled architecture geometry [11,12]. For instance, when *para*-diiodotetrafluorobenzene, or its dibromo analogue, self-assemble with linear XB acceptors, e.g., 4,4’-dipyridyl, linear infinite chains are formed; when *meta* or *ortho* isomers are used, zig-zag infinite chains are obtained with angles along the chain close to 120° and 60°, respectively [13,14,15,16,17,18]. Iodoaromatics are usually good XB donors and this is particularly true when the aromatic moiety bears electron withdrawing groups [2], and it can be expected that 1,3,5-trifluorotriiodobenzene (**1**) has a tendency to work as a tritopic XB donor after a trigonal geometry and to afford (6,3) networks when interacting with bi- or tritopic acceptors [19,20]. A search in the Cambridge Structural Database (CSD) confirms that this may be the case [21,22,23]. On the other hand, calculations have shown that, as a consequence of the charge transfer component of the interaction [19], the XB donor ability of the iodine atoms of **1** decreases when the number of XBs the tecton (**1**) is already involved in is increased [20]. In other words, the formation of the third XB is energetically less favored than the second, and the formation of the second less favored than the first one [24]. Indeed, the number of XBs given by the XB donor **1** with usual acceptors (e.g., N atoms and anions) can be two or even one [25,26,27], and it has been argued that the preferential formation of infinite chains (1D nets) rather than honeycomb systems (2D nets) may also be associated with steric reasons, i.e., the problem to fill the potential empty space encircled by the hexagonal frame. Moreover, the number of XBs formed by a given acceptor on interaction with **1** may vary, e.g., if solvated co-crystals are formed [28,29]. Moreover, when a series of compositionally related co-crystals is obtained on assembly of **1** with a series of salts wherein the cation is the same and the anion changes, the number of XBs formed by the different anions may vary or remain unchanged and the same may happen for the topology of the obtained supramolecular anions [18,21]. For instance, **1** and tetra-*n*-butylammonium iodide [21], or bromide and chloride [30] give 2D systems wherein both the XB donor and acceptors are tritopic; however, when tetra-*n*-butylammonium thiocyanate is used [22], a 1D system is formed wherein **1** is tritopic and the anion either mono- or bitopic.

**Scheme 1:**
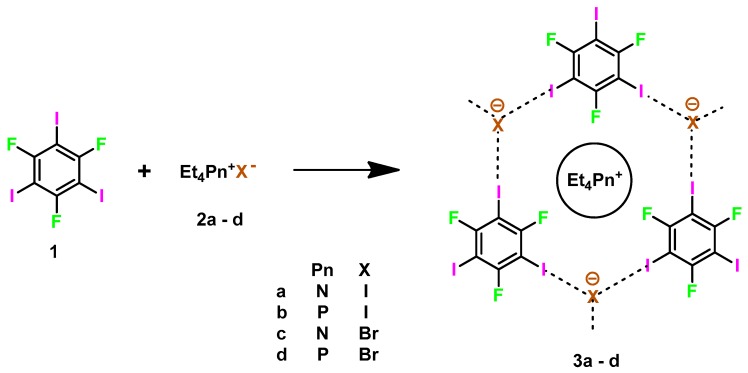
The formation of the two species (i.e., iodobenzene derivative **1** and salts **2a**–**d**) and three component (i.e., **1**, the cation, and the anion from salts **2a**–**d**) co-crystals **3a**–**d** via self-assembly of 1,3,5-trifluorotriiodobenzene (**1**) with onium halides **2a**–**d**.

It thus seems that the connectivity and topology landscape of halogen bonded adducts given by the donor **1** is quite diversified and not easy to predict, and this is particularly true when anionic acceptors are used. We have already reported a series of adducts between **1** and iodide salts where both **1** and the iodide anion are tritopic and the systematic change of the cation showed that the cation size plays a major role in enabling the formation of (6,3) networks [21]. It is required that cations fit in the cavity encircled by the hexagonal frames of the nets and the upper metric limit for the onium cation, enabling the I∙∙∙I^−^ supramolecular synthon to form (6,3) networks that lie between the dimensions of tetra-*n*-propyl- and tetra-*n*-butylammonium cations. For instance, the self-assembly of **1** with Et_4_N^+^I^−^ or Et_4_P^+^I^−^ (**2a**,**b**) affords co-crystals **3a**,**b** where both **1** and the iodide ions are tritopic and form (6,3) networks where the two modules alternate at the network nodes (Scheme 1); the same happens with *n*-Pr_4_N^+^I^−^ but not with *n*-Bu_4_N^+^I^−^.

**Figure 1:**
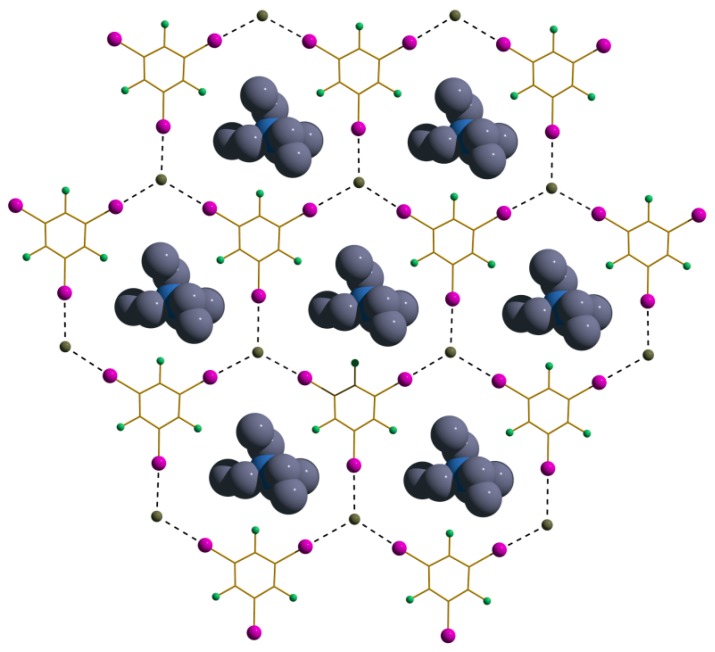
Partial representation of one honeycomb network of **3c**. The Et_4_N^+^ cations, sitting at the center of the hexagonal cavities, are represented with space-filling models. The XBs are black dotted lines. Color code: C: grey; N: blue; I: purple; F: light green; Br: olive.

As a part of an ongoing project aimed at changing the composition of multi-component and self-assembled systems, while maintaining unmodified their supramolecular characteristics [31], we were interested in identifying cases where the change of the anion in **3a**,**b** preserves all the supramolecular characteristics described above, and decided to co-crystallize **1** with Et_4_N^+^Br^−^ and Et_4_P^+^Br^−^ (**2c**,**d**) (Scheme 1). We reasoned that the formation of (6,3) networks **3c**,**d** from **1** and **2c**,**d** is quite likely while the hexagonal frames having bromide anions at three of their vertexes should be smaller than analogous frames having iodide anions (I∙∙∙Br^−^ bonds are typically shorter than I∙∙∙I^−^ bonds), the used onium cations would comfortably fit also in the smaller cavities formed by bromide anions as tetraethyl onium cation is safely below the cation upper size limit for the formation of (6,3) nets based on the I∙∙∙I^−^ supramolecular synthon. Here, we report that the connectivity and the topology in the adducts **3c**,**d** (afforded by **1** and **2c**,**d**) is the same as in **3a**,**b** (Figure 1).

The number of XBs given by the donor **1** and by iodide and bromide acceptors remains unchanged in the four co-crystals **3a**–**d**, and the same holds for the topology of the respective supramolecular anions, as the contraction of the hexagonal frames size resulting from bromide for iodide substitution has been tolerated, and cations invariably sit in the hexagonal cavities.

## 2. Results and Discussion

Whitish crystalline solids (**3c**,**d**) were obtained on slow evaporation at room temperature of equimolar solutions of **1** and tetraethylammonium or -phosphonium bromide (**2c** or **2d**, respectively) in CH_2_Cl_2_/methanol mixtures. The melting points of these solids were quite sharp and in between those of starting compounds, suggesting that well-defined chemical species had been obtained rather than physical mixtures. IR spectra showed the presence of peaks of both C_6_F_3_I_3_ and the onium moieties, only minor differences in peak intensities and wave numbers were observed with respect to pure starting compounds.

^1^H and ^19^F NMR analyses in the presence of bis(2,2,2-trifluoroethyl) ether as internal standard for peaks integration [32] revealed that the starting compound ratio is 1:1.

Single crystals X-ray analyses of **3c**,**d** confirmed the starting compounds ratio established via the NMR technique and revealed that both compounds crystallize in the trigonal space group *R*$\bar{3}c$ **1** and the bromide anions act as tritopic trigonal XB donors and acceptors, respectively, leading to 2D anionic networks with (6,3) topology. The bromide ions and iodobenzene moiety **1** alternate at the nodes of the net and Et_4_N^+^ or Et_4_P^+^ cations are accommodated in the center of the hexagonal space.

Two different I∙∙∙Br^−^ contacts and C-I∙∙∙Br^−^ angles are present in both **3c** and **3d**, and the respective values are quite similar in the two structures. I2∙∙∙Br1 distances are 329.4(5) and 330.2(5) pm in **3c** and **3d**, respectively, while I1∙∙∙Br1 distances are slightly longer (341.6(3) and 343.8(4) pm). These values correspond to normalized contacts (N_c_) that are quite small [33] (they are in the range 0.84–0.87), suggesting that XBs in these structures are fairly strong. This is even more notable [19,20] if we consider that the electron donor ability of the bromide and the electron acceptor ability of the iodofluorocarbon are split over three XBs. Consistent with this strength, the two different C-I∙∙∙Br angles present in **3c**,**d** are almost linear (they span in a value range of 172.87(5)–179.88(5)°). The hexagonal motifs of the 2D honeycomb networks are slightly distorted due to the deviation of the I∙∙∙Br^−^∙∙∙I angles (they vary between 117.68(1)° and 124.62(1)°) from the ideal 120° for a symmetric trigonal coordination.

**Figure 2:**
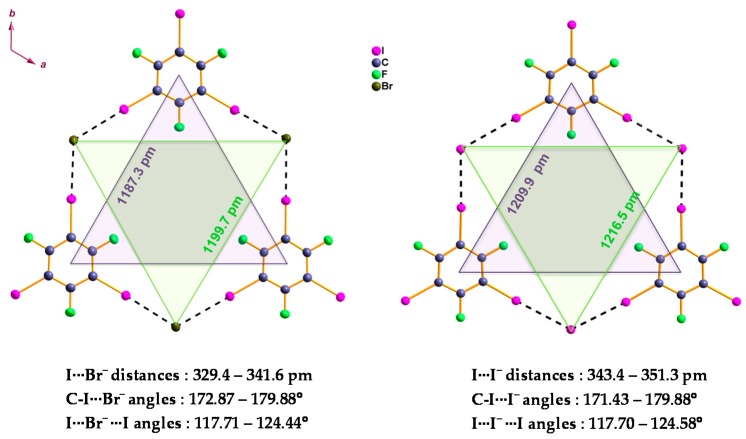
A view along the crystallographic *c* axis of one hexagonal frame of **3c** (**left**) compared to the corresponding frame for **3a** (**right**). Semi-transparent triangles connect the naked Br^−^ or I^−^ ions (greenish) and the centers of the phenyl rings of **1** (purple).

**Figure 3:**
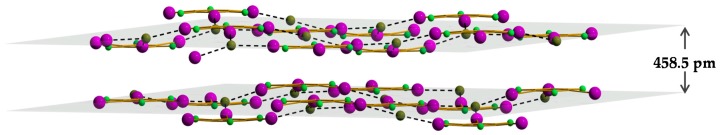
Two supramolecular anionic layers in the crystal structure of **3d**. Semitransparent planes are the mean square planes through the anions of a layer. Color codes as in Figure 1; brown lines are the covalent bonds; black dotted lines are XBs; Et_4_P^+^ cations have been omitted for clarity.

All these features nicely parallel those in corresponding nets formed by iodide anions and a comparison between the hexagonal frames of **3c** and **3a** (refcode CIZRUZ) [21] is depicted in Figure 2. The dimensions of the hexagonal cavities are estimated by the sides of the two triangles; one is connecting the naked Br^−^ or I^−^ ions and the other one the centroids of the phenyl rings of **1**. Iodide anions are larger than bromide anions and I∙∙∙ I^−^ halogen bonds are longer than the I∙∙∙ Br^−^ ones, resulting in hexagons with slightly greater dimensions in the iodide based nets. Despite this, the honeycomb topology is sustained, consistent with the fact that its formation is critically dependent on the cation’s sitting in the internal space of the hexagonal frame and with the fact that the critical cation size was found between the dimensions of *n*-Pr_4_N^+^ and *n*-Bu_4_N^+^ in honeycomb nets formed by iodide anions. The ability of the hexagonal frames formed by bromide anions to nicely accommodate Et_4_N^+^ and Et_4_P^+^ cations in **3c**,**d** is confirmed by the fact that the 2D supramolecular anions are quite flat (Figure 3), while they become undulated when the frame/cation mismatch forces the cations to protrude out of the hexagons [21].

The cations’ nature affects the distance between two adjacent anionic layers as well; specifically, the larger the cation is, i.e., the more it protrudes out of the hexagons, the greater the interlayer separation becomes [34]. The distance between the two adjacent layers is 437.0(1) pm in **3c** and is slightly greater in **3d** (458.5(1) pm). This trend has also been observed between **3a** and **3b** (interlayer separations are 452.9 and 460.1 pm, respectively) and is probably due to the fact that a phosphonium cation is larger than the corresponding ammonium cation. The interlayer separations in **3c**,**d** are smaller than the separation of the two supramolecular anionic nets in the co-crystal formed by the triiodobenzene derivative **1** with tetra-*n*-propylammonium iodide (554.9 pm), the largest tetraalkylammonium iodide that affords a honeycomb net upon self-assembly with **1**.

Short contacts exist between the fluorine atoms of **1** and the partially positive hydrogen atoms of the methylene groups bound to the nitrogen and phosphorous atoms of **3c**,**d** (Figure S1), and this suggests that onium cations potentially serve as templating agents for the formation of the hexagonal frames. These contacts can be considered as C-H∙∙∙F-C hydrogen bonds and may play a role in assisting the self-assembly of the hexagonal anionic frames. This possibility is supported by the presence of similar HBs in related honeycomb nets [21]. The templating role of the cation is backed by the fact that, in the honeycomb network formed by triethyl-chloromethylammonium chloride [25], the XB donor **1** is pinned in its position by the I∙∙∙Cl^−^ XBs forming the supramolecular anion, by the H∙∙∙F HBs involving the “acidic” methylene groups, and by a further XB wherein the chlorine of the N^+^CH_2_Cl moiety is the XB donor and the belt of the iodine of **1** is the acceptor.

**Figure 4:**
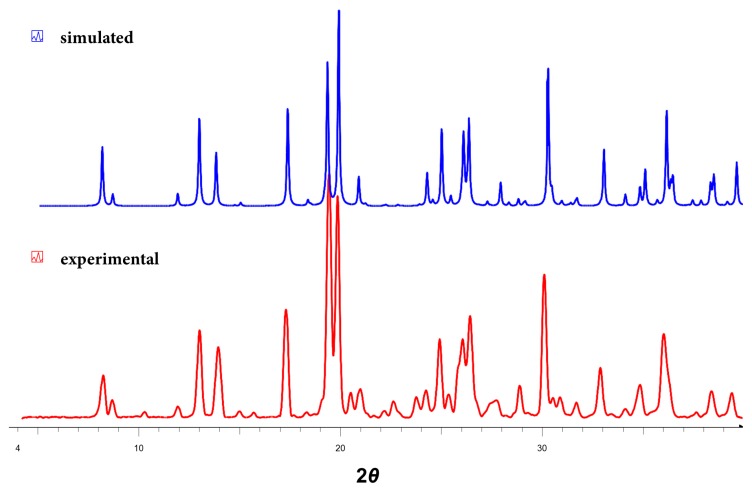
Powder X-ray Diffraction patterns of **3d**.

In order to confirm that metric aspects enable massive self-assembly of **1** and **3c**,**d** into honeycomb nets, powder X-ray diffraction (pXRD) analyses were carried out for **3c** (Figure S5) and **3d** (Figure 4). Experimental patterns nicely match patterns simulated from single crystal analyses, and phase purity of the crystallized compounds is thus proven.

**Figure 5:**
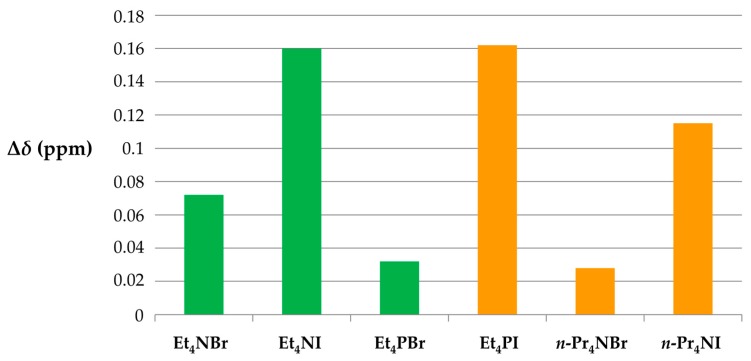
Variation of ^19^F NMR chemical shift of 1,3,5-triidotrifluorobenzene (**1**) upon interaction with some onium bromides and iodides when 10 equivalents of the onium salt was added to a 5 mM solution of **1** in deuterochloroform.

Finally, ^19^F NMR experiments were performed to assess the XB presence between **1** and **2c**,**d** in solution (Figure 5). Small upfield shifts were observed for the signal of **1** upon the addition of different onium salts, with the chemical shift changes ranging from 0.028 to 0.162 ppm (Table S1). For a given cation, bromide salts afforded smaller chemical shift changes than iodide salts in all cases, consistent with observations reported for other haloperfluorocarbons/halide adducts in solution [35].

## 3. Materials and Methods

### 3.1. General Information

The starting materials 1,3,5-trifluorobenzene, tetraethylammonium bromide, and tetraethylphosphonium bromide were purchased from Sigma-Aldrich and used without further purification. Melting points were determined on a Reichert instrument by observing the melting process through an optical microscope. ATR-FTIR spectra were obtained using a Nicolet Nexus FTIR spectrometer. Peaks frequencies, given in wave numbers, were rounded to 1 cm^−1^ using automatic peak assignment.

### 3.2. Procedures and Compound Characterization

#### 3.2.1. Preparation of 1,3,5-Triiodo-2,4,6-trifluorobenzene (**1**) [36]

KI (7.14 g, 43.56 mmol) was added slowly to a stirred mixture of periodic acid (3.30 g, 14.50 mmol) in concentrated H_2_SO_4_ (20 mL) at 0 °C. The obtained dark mixture was cooled with an ice bath while 1,3,5-trifluorobenzene (1.0 mL, 9.68 mmol) was added over 25 min. The mixture was heated to 70 °C for 4 h, then cooled to room temperature, poured on ice, and extracted with diethyl ether (3 × 50 mL). The collected organic phases were washed with sat. Na_2_S_2_O_3_ and water, then dried with Na_2_SO_4_. After evaporation of the solvent, **1** was recovered as a pure white powder in 80% yield. ^19^F NMR (CDCl_3_, 235 MHz): δ −69.90 ppm; m.p.: 158–159 °C; FT-IR (KBr pellet, cm^−1^): 1561, 1401, 1325, 1048, 704, 652.

#### 3.2.2. Preparation of Co-Crystal **3c**

Co-crystals of **3c** were formed by dissolving equimolar amounts of 1,3,5-trifluorotriiodobenzene and tetraethylammonium bromide in a mixture of CH_2_Cl_2_/ΜeOH (1:1) and after slow isothermal evaporation of the solvents at room temperature. m.p.: 240–242 °C. FT-IR (KBr pellet, cm^−1^): 2982, 1563, 1473, 1458, 1394, 1184, 1036, 1005, 794, 709.

#### 3.2.3. Preparation of Co-Crystal **3d**

A procedure similar to that used for **3c** was employed. m.p.: 238-241 °C. FT-IR (KBr pellet, cm^−1^): 2905, 1561, 1458, 1393, 1331, 1267, 1036, 779, 709.

### 3.3. Single Crystal Structure Determination

The single crystal X-ray diffraction measurement of the **3c**,**d** was conducted on a Bruker SMART APEX CCD area detector diffractometer, equipped with a Bruker *KRYOFLEX* low temperature device, graphite monochromator, Mo *K*α radiation (λ = 0.71069 Å) at 123 K. Cell refinement and data reduction were performed with a Bruker SAINT [37]. The structures were solved with SHELXS [38] and refined with SHELX-97 [38]; absorption correction was performed based on a multi-scan procedure using *SADABS* [37]. Further crystallographic details of the structures in this paper are reported in Table S2.

### 3.4. ^19^F NMR Experiments

^19^F NMR spectra were recorded on a Bruker ADV 500 spectrometer at 25 °C, CDCl_3_ was used as solvent and CFCl_3_ as internal standard. Δδ values reported in Figure 4 for ^19^F NMR chemical shift of 1,3,5-trifluorotriiodobenzene (**1**) were obtained upon the addition of 10 equivalents of the onium halides (*n*-Pr_4_N^+^Br^−^, Et_4_N^+^Br^−^, Et_4_P^+^Br^−^, *n*-Pr_4_N^+^I^−^, Et_4_N^+^I^−^, and Et_4_P^+^I^−^) to 5 mM solutions of **1** in CDCl_3_: δ_F_ (ppm, 5 mM sol. of **1** in CDCl_3_) = −69.90; Δδ_F_ (ppm) = δ_5 mM **1**_ − δ_5 mM **1** + 10 eq. onium halide_).

### 3.5. Powder X-ray Diffraction Analyses

The crystalline powder material of co-crystals was packed on borosilicate glass slides and the data sets were collected on a Bruker D8 instrument at 293 K. The measurements were made in Bragg–Brentano geometry using Johansson monochromator to produce pure CuKα_1_ radiation (1.5406 Å; 45 kV, 30 mA) and a step–scan technique in the 2θ range of 4–40°. The data were acquired from a spinning sample by an X´Celerator detector in continuous scanning mode with a step size of 0.0167° using a sample dependently counting times of 90 s per step. The comparison of simulated and experimental PXRD pattern confirms the structural uniformity of bulk co-crystal powders.

## 4. Conclusions

In general, different halides can assemble different structures under control of electrostatic interactions, metal coordination, and HB [39], and the same holds when XB is the driving force of the self-assembly processes [40,41]. Different halides can present different coordination spheres, and bromide for iodide substitution in heteromeric multicomponent systems (**3**) is expected to give rise to XBs and electrostatic attraction between opposite ions (the two strongest interactions in co-crystals **3a**–**d**) endowed with strengths that are quite different [42]. The triiodobenzene derivative **1** can work as a mono-, bi-, or tritopic XB donor [43], and bromide anions frequently function as di-, tri-, or tetratopic acceptors [44], but such anions can also present other XBs in their first coordination sphere, as they have been reported to act even as octatopic acceptors [45]. In the systems described here, the tetraethyl onium cations enable the assembly of three units of **1** around a bromide anion and combined matching, both at steric and electronic levels, of all the components of **3a**–**d** allows **1** to work as a planar and trigonal tecton in the XB driven self-assembly of supramolecular halide networks. This behavior recalls the HB driven self-assembly processes wherein trimesic acid or 1,3,5-triazine derivatives function as planar and threefold tectons.

The supramolecular similarity of **3a**–**d** indicates that, metric requirements being fulfilled, the ability of **1** to function as a tritopic XB donor is robust enough to elicit the tritopic acceptor potential of bromide anions, to tolerate non-minor differences in the strength of interactions driving the co-crystals self-assembly, and to afford the honeycomb nets. The similarity of **3a**–**d** gives rise to further examples wherein the mutual induced fit among the different components of a heteromeric co-crystal elicits their respective ability to act as tritopic tectons and affords honeycomb nets, with either bromide or iodide anions at the nodes [21]. The use of this heuristic principle in the design of other multi-component systems assembly with the same topology but with different compositions is under study.

## Supplementary Materials

The following are available online: CCDC 1576571 and 1576572 contain the supplementary crystallographic data for this paper. These data can be obtained free of charge via www.ccdc.cam.ac.uk/data_request/cif, or by emailing data_request@ccdc.cam.ac.uk, or by contacting The Cambridge Crystallographic Data Centre, 12, Union Road, Cambridge CB2 1EZ, UK; fax: +44 1223 336033.
