# Supplementary material for: Connectivity and Topology Invariance in Self-Assembled and Halogen-Bonded Anionic (6,3)-Networks
Source: Molecules. 2017 Nov 24;22(12):2060. doi: 10.3390/molecules22122060 (PMC6149883; doi:10.3390/molecules22122060)
Supplement: Supplementary file 1 [file molecules-22-02060-s001.pdf]

Supplementary Information

Connectivity and Topology Invariance in Self-Assembled and Halogen-Bonded Anionic (6,3)-Networks

Franck Meyer, Tullio Pilati, Konstantis F. Konidaris, Pierangelo Metrangolo, Giuseppe Resnati*

Laboratory of Nanostructured Fluorinated Materials (NFMLab), Department of Chemistry, Materials, and Chemical Engineering "Giulio Natta", Politecnico di Milano, Via L. Mancinelli 7, 20131 Milano, Italy

Table S1. Variation of ^{19}F NMR chemical shift of 1,3,5-triidotrifluorobenzene (**1**) upon interaction with some onium bromides and iodides when 10 equivalents of the onium salt was added to a 5 mM solution of **1** in deuterochloroform.

**1**	**1+*n*-Pr$_4$NBr**	**1+Et$_4$PBr**	**1+Et$_4$NBr**	**1+*n*-Pr$_4$NI**	**1+Et$_4$NI**	**1+Et$_4$PI**
-69.900 ppm	-69.928	-69.932	-69.972	-70.015	-70.060	-70.062
**$\Delta\delta_F$** (ppm)[a]	0.028	0.032	0.072	0.115	0.160	0.162

[a] $\Delta\delta_F$ (ppm) = $\delta_{5\ mM\ \mathbf{1}} - \delta_{5\ mM\ \mathbf{1} + 10\ eq.\ onium\ halide}$).

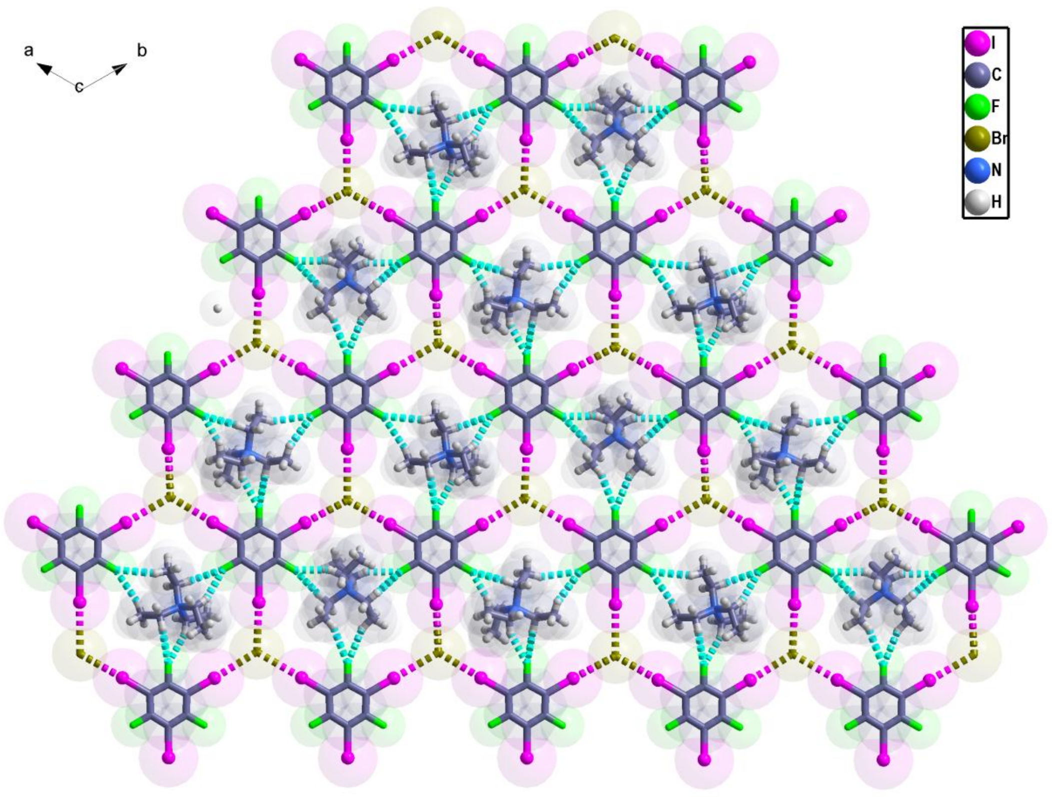

Figure S1. A 2D sheet in the crystal structure of **3c** showing weak hydrogen bonds (turquoise dotted lines) occurring between the fluorine atoms of **1** and the hydrogen atoms of **2c**. A semitransparent space-filling model is shown as superimposition of all atoms.

Table S2. Crystallographic data and structure refinement parameters for **3c** and **3d.**

**Compound**	**3c**	**3d**
Formula	$C_{14}H_{20}BrF_3I_3N$	$C_{14}H_{20}BrF_3I_3P$
Molecular weight	719.92	736.88
Temperature (K)	123	123
Crystal system	Trigonal	Trigonal
Space group	$R\bar{3}c$	$R\bar{3}c$
a (Å)	20.424(3)	20.409(3)
c (Å)	26.218(4)	27.511(4)
α (°), β (°)	90.00	90.00
γ (°)	120.00	120.00
Volume (Å^3)	9471(2)	9924(2)
Z	18	18
Crystal size (mm^3)	0.33 × 0.31 × 0.13	0.14 × 0.09 × 0.05
μ (mm^{-1})	6.37	6.15
F(000)	5976	6120
No. of measured, independent and observed reflections	39029, 4371, 3585	26542, 4531, 3316
θ_{min}, θ_{max} (°)	1.9, 34.5	1.9, 34.5
$R_{\_ave}$	0.026	0.034
wR_{2_all}, wR_{2_obs}	0.078, 0.076	0.091, 0.087
GOOF	1.084	1.004
$\Delta\rho_{min}$, $\Delta\rho_{max}$ (e Å^{-3})	-0.68, 1.86	-0.85, 2.13
CCDC number	1576571	1576572

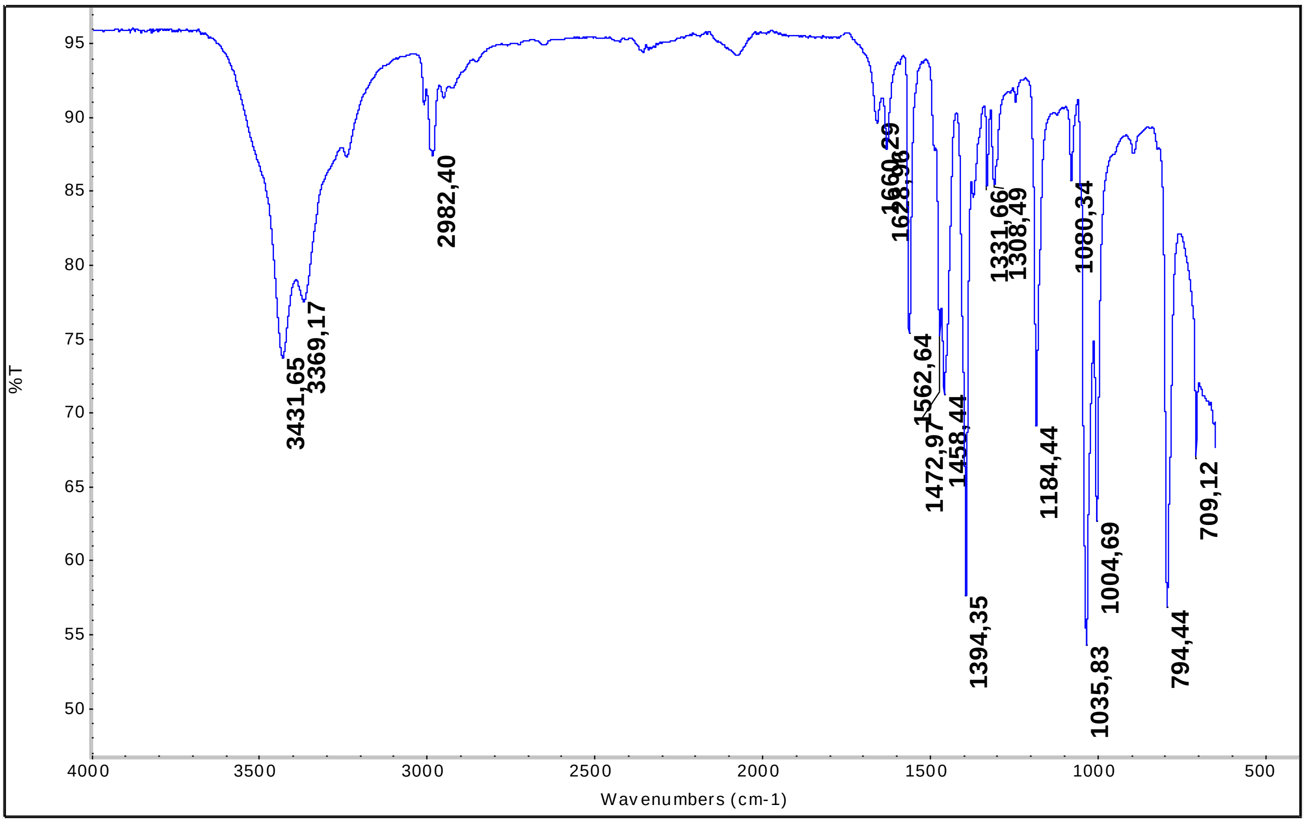

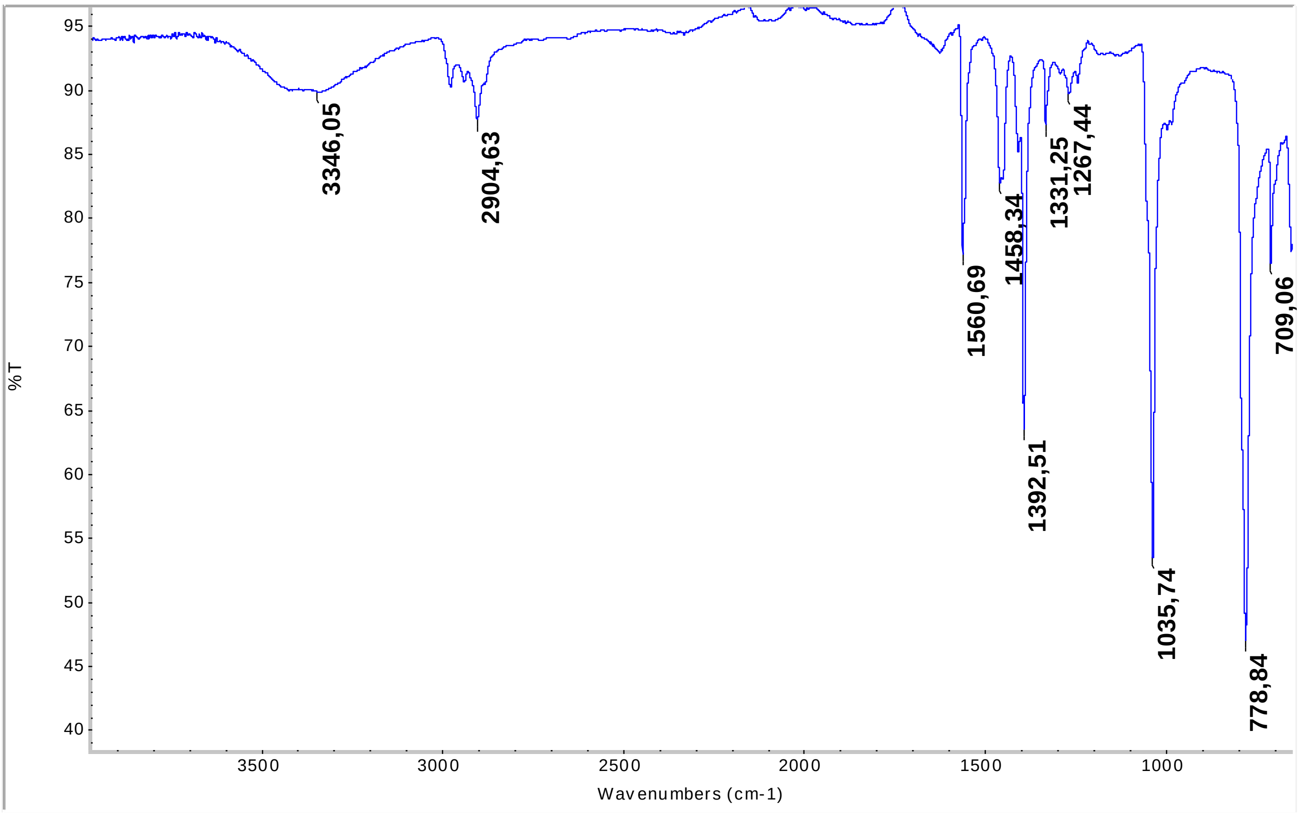

Figure S2. FT-IR spectra of compounds **3c** (top) and **3d** (bottom).

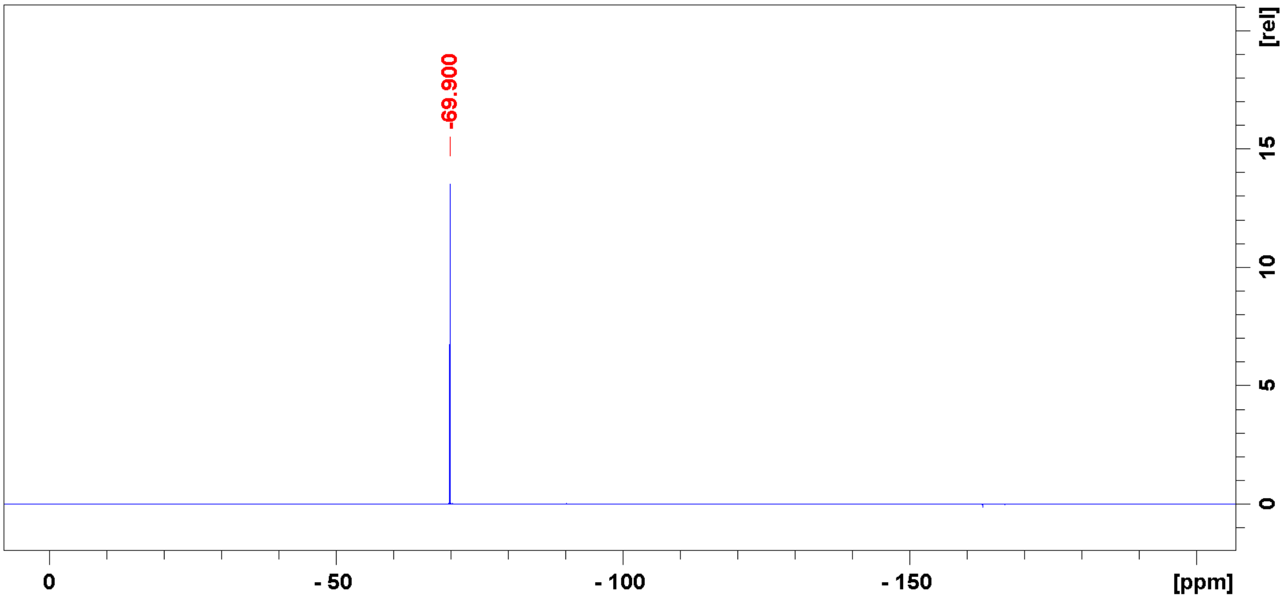

Figure S3. ^{19}F-NMR spectrum of 1,3,5-trifluorotriiodobenzene (**1**) in $CDCl_3$.

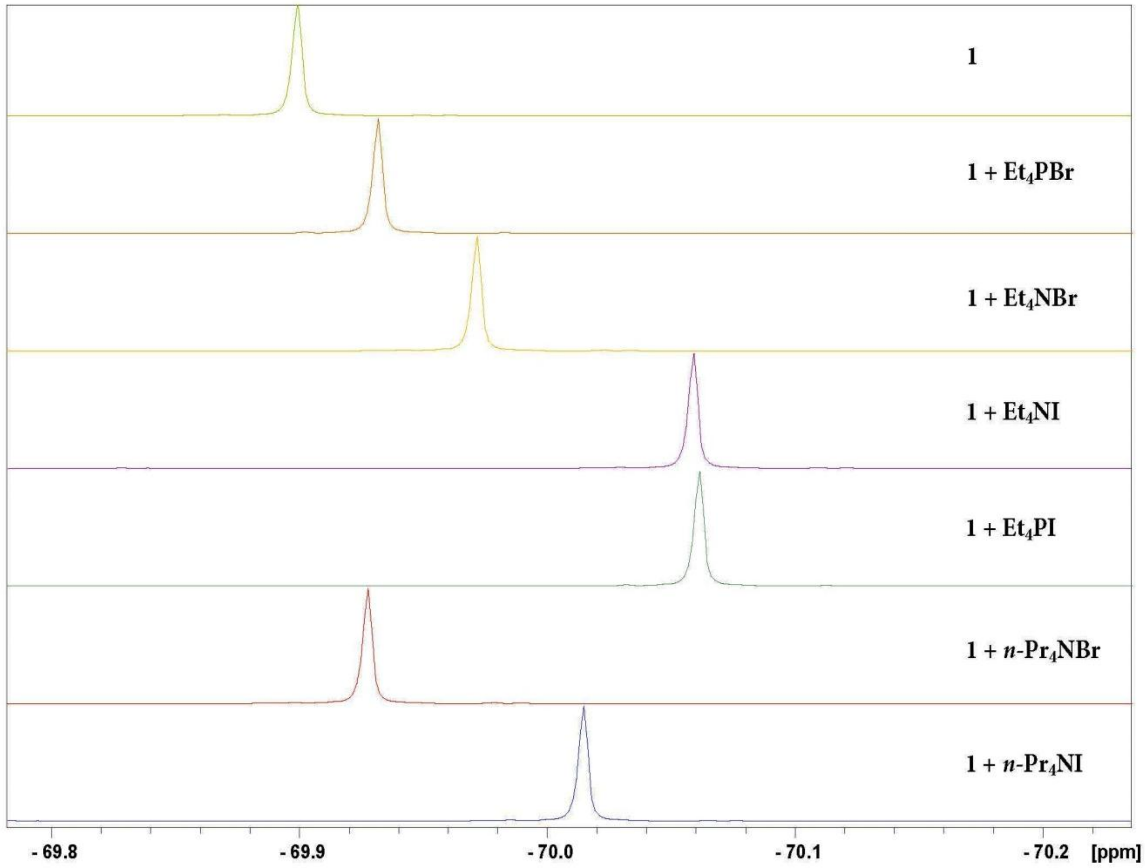

Figure S4. ^{19}F-NMR spectra of 1,3,5-trifluorotriiodobenzene (**1**, 0.05 mM solution in $CDCl_3$) upon addition of ten equivalents of the respective onium salt.

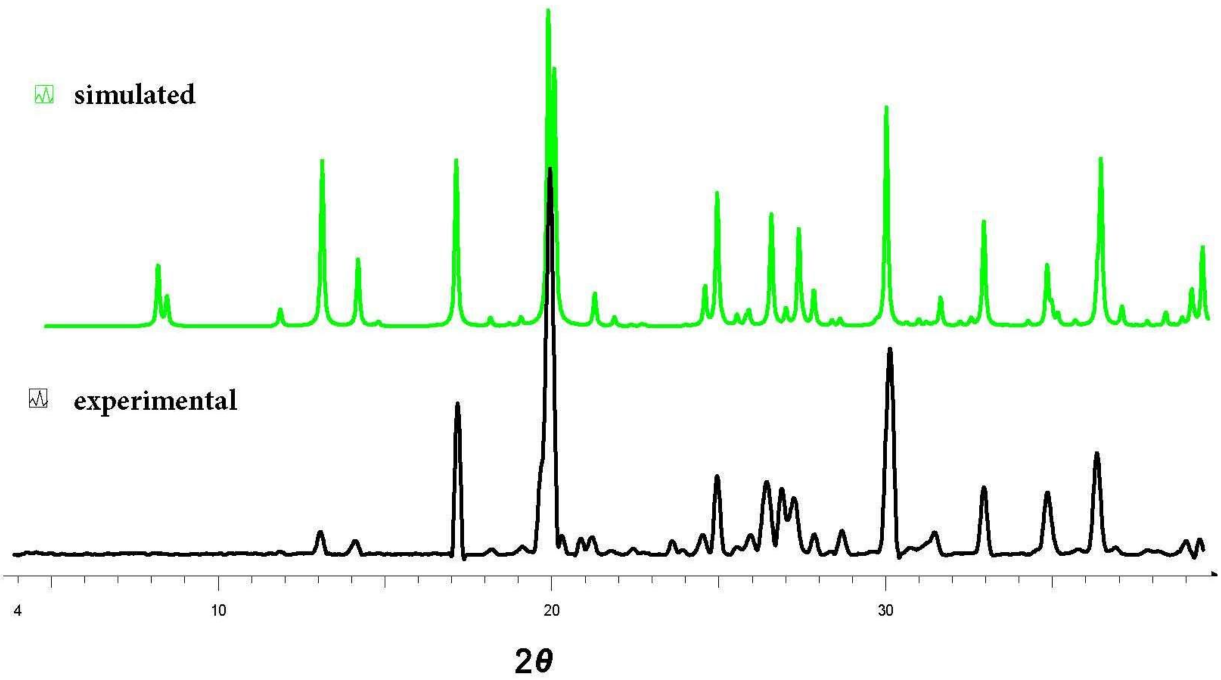

Figure S5. Powder X-ray Diffraction pattern of compound **3c**.
